# Patient and care partner expectations and emotional experiences of lecanemab: A social media listening study

**DOI:** 10.1002/alz70860_101001

**Published:** 2025-12-23

**Authors:** Monica K Crane, Lee Watson, Amanda Blackwood, Pippa Brown, Vicky Britton, Alison Bulkley, Marc DeCongelio, Craig Plauschinat, Feride H Frech, Hideki Toyosaki

**Affiliations:** ^1^ Genesis Neuroscience Clinic LLC, Knoxville, TN, USA; ^2^ University of Tennessee‐Knoxville, Knoxville, TN, USA; ^3^ Genesis Neuroscience Clinic, Knoxville, TN, USA; ^4^ Oracle Life Sciences, London, United Kingdom; ^5^ Oracle Life Sciences, Austin, TX, USA; ^6^ Eisai, Inc, Nutley, NJ, USA; ^7^ Eisai Inc., Nutley, NJ, USA

## Abstract

**Background:**

Patients and care partners are in unique positions to provide valuable perspectives on Alzheimer's disease (AD). The objective of this study was to better understand the expectations and emotional experiences of AD patients and care partners with lecanemab, utilizing social media posts

**Method:**

This retrospective study examined lecanemab‐specific public social media data from AD patients and care partners based in the U.S. between January 2023 and October 2024. Using Brandwatch, Sprinklr and Buzzsumo, 468 lecanemab‐related posts were collected via detailed keyword strings which identified posts using first‐person or familial related‐related language. Key themes were identified qualitatively through a systematic review by two researchers, with a third overseeing the process to ensure rigor and reduce bias. Quantitative insights were based off keyword frequency.

**Result:**

Using the keyword “lecanemab”, we retrieved posts from the social media sites Reddit (64% of posts), X.com (20%), digital media (12%), forums (3%), and Facebook/Quora (1%). “Risk” was mentioned in 28% of lecanemab posts. “Fear” was referenced in 8% of posts; specifically fear of side effects as a key reason for declining the treatment. References to “hope” were mentioned in 23% of posts and were strongly tied to perceived efficacy and the prospects or experience of disease slowing. “Family” and “more time” were mentioned across 14% and 5% of posts respectively, seeking “more time”. The themes included the desire for treatment to lengthen the amount of time family members can spend with those affected with AD in a stage perceived as relative normality before routine and behaviors are severely disrupted.

**Conclusion:**

The emotional themes of risk, hope, family, fear, and more time are interwoven throughout social media with regards to lecanemab. Hope for treatment to delay disease progression was the strongest theme, while risk and fear are alluded as reasons for declining treatment. The results demonstrate the importance of social media as an engagement tool of current topics in dementia research and reveal areas of potential for increased engagement. Improved understanding of the expectations and emotional experiences of treatment may assist in patient‐centered treatment decision‐making and identify unmet patient needs.

